# Characteristics of PM_2.5_-Bound Polycyclic Aromatic Hydrocarbons and Nitro-Polycyclic Aromatic Hydrocarbons at A Roadside Air Pollution Monitoring Station in Kanazawa, Japan

**DOI:** 10.3390/ijerph17030805

**Published:** 2020-01-28

**Authors:** Wanli Xing, Lulu Zhang, Lu Yang, Quanyu Zhou, Xuan Zhang, Akira Toriba, Kazuichi Hayakawa, Ning Tang

**Affiliations:** 1 Graduate School of Medical Sciences, Kanazawa University, Kanazawa 920-1192, Japan; xingwanli@stu.kanazawa-u.ac.jp (W.X.); zhang-lulu@stu.kanazawa-u.ac.jp (L.Z.); veronicayl@stu.kanazawa-u.ac.jp (L.Y.); zhouquanyu@stu.kanazawa-u.ac.jp (Q.Z.); zhangxuan@stu.kanazawa-u.ac.jp (X.Z.); 2 Institute of Medical, Pharmaceutical and Health Sciences, Kanazawa University, Kanazawa 920-1192, Japan; toriba@p.kanazawa-u.ac.jp; 3 Institute of Nature and Environmental Technology, Kanazawa University, Kanazawa 920-1192, Japan; hayakawa@p.kanazawa-u.ac.jp

**Keywords:** polycyclic aromatic hydrocarbons, nitro-polycyclic aromatic hydrocarbons, roadside sampling, traffic emission, diagnostic ratios

## Abstract

Polycyclic aromatic hydrocarbons (PAHs) and nitro-PAHs (NPAHs) in PM_2.5_ samples were collected at a roadside monitoring station in Kanazawa, Japan, in every season from 2017 to 2018. Nine PAHs and five NPAHs were determined using high-performance liquid chromatography with fluorescence detection and chemiluminescence detection, respectively. The mean concentrations of PAHs and NPAHs were highest in winter and lowest in summer. Fluoranthene and pyrene were the dominant PAHs and 1-nitropyrene was the dominant NPAH in all seasons, and these compounds were mainly emitted by diesel vehicles. The concentration ratio of benzo(*a*)pyrene (BaP) to benzo(*ghi*)perylene (BgPe) ((BaP)/(BgPe)) and of indeno(1,2,3-*cd*)pyrene (IDP) to the sum of IDP and benzo(*ghi*)perylene (BgPe) ((IDP)/((IDP)+(BgPe0) might still be useful indicators for identifying traffic emission sources today. Moreover, our results showed that the carcinogenic risk in all seasons was below the acceptable limit set by the WHO.

## 1. Introduction

Ambient particulate matter (PM) is a well-known atmospheric pollutant and greatly influences air quality and human health [1,2]. PM with an aerodynamic diameter ≤ 2.5 μm (PM_2.5_) is considered more harmful because it can be enriched in toxic components and respirable particulates that can penetrate deeply into the lungs [3]. PM_2.5_ can contain a variety of substances, including polycyclic aromatic hydrocarbons (PAHs), which are organic compounds composed of multiple aromatic rings [4]. PAHs pose health risks due to their potential toxicity, including carcinogenicity and mutagenicity. Several nitro-PAHs (NPAHs), such as 1-nitropyrene (1-NP), exhibit higher toxicity than their parent PAHs due to their direct mutagenicity [5]. The International Agency for Research on Cancer (IARC) has classified benzo(*a*)pyrene (BaP) as a Group 1 carcinogen (carcinogenic to humans) and 1-NP as a Group 2A carcinogen (probably carcinogenic to humans) [6].

PAHs originate from fossil fuel and biomass combustion [7]. Some NPAHs are directly emitted from combustion sources [5], and several NPAHs are formed from the reactions of their parent PAHs with hydroxyl (OH) and nitrate (NO_3_) radicals in the presence of nitrogen oxides (NO_x_) [8,9]. Because individual congeners are produced in similar ways, PAHs and NPAHs are often considered mixtures. In addition, the form of PAHs and NPAHs largely depends on the combustion temperature and fuel type [10,11]. Low-temperature combustion processes, such as coal and biomass combustion, mainly produce 2-to-4-ring congeners, while higher molecular weight congeners mainly originate from high-temperature processes, such as those in vehicle engines. Gasoline vehicles are the major source of 5-to-6-ring congeners, while diesel vehicles are the major source of lighter congeners [12]. At present, the emission source identification of PAHs and NPAHs in ambient air makes use of diagnostic ratios (DRs) or receptor models, such as the positive matrix factorization (PMF) method [13,14]. However, DRs can identify only the main source of PAHs and NPAHs, and PMF requires a large number of samples and other components of PM_2.5_ to decrease the uncertainty. In addition, after being released into ambient air, PAHs and NPAHs are altered on the basis of their physical and chemical properties. Therefore, it is important to explore the characteristics of particulate PAHs and NPAHs emitted directly from their source to improve the effectiveness and accuracy of source analysis.

The Japan Society for Atmospheric Environment (JSAE) reported that since the 1980s, the main contributor to air pollution in commercial cities in Japan has changed from factories to traffic emissions [15]. According to the statistical information on automobile ownership released by the Japan Automobile Service Promotion Association (JASPA), the total number of vehicles in Japan reached 81.8 million with an average of 648 vehicles per 1000 inhabitants by March, 2019. (https://www.jaspa.or.jp/member/data/owned.html) Our previous studies indicated that the dominant source of atmospheric PAHs and NPAHs was traffic emissions, especially diesel-engine vehicles in commercial cities in Japan, such as Tokyo, Sapporo and Kanazawa [16,17]. In response to the negative impact from traffic emissions to air quality, the Japan Ministry of Land, Infrastructure, Transport and Tourism has gradually increased controls over the levels of NO_x_ and PM emitted from new vehicles since 1989 [18]. As a result, the concentrations of PAHs and NPAHs from traffic emissions in the urban areas of Kanazawa decreased 69.8% and 88.6%, respectively, from 1999 to 2010 [19]. Many studies have analysed the concentrations of PAHs and NPAHs in traffic emissions in Kanazawa [16,17,19,20]. However, these studies concentrated on variations in concentration, and there is a lack of analysis on the composition characteristics of PAHs and NPAHs directly emitted from vehicles. Recently, a portable emission measurement system was developed and widely used to measure the PAH and NPAH emissions from on-road vehicles [21,22]. However, these results failed to reflect the mixed emission characteristics of vehicles with different types and engine loadings.

In this study, PM_2.5_ was collected at a roadside air pollution monitoring station in Kanazawa, Japan. Our aims were to analyse the compositions of and seasonal variations in PM_2.5_-bound PAHs and NPAHs emitted from vehicles on the road, to compare the measured and previous DRs and to identify ratios that indicate mixed traffic sources. The lifetime cancer risk (LCR) of PAHs and NPAHs in PM_2.5_ were also evaluated at this site.

## 2. Materials and Methods

### 2.1. PM_2.5_ Sampling

**Figure 1:**
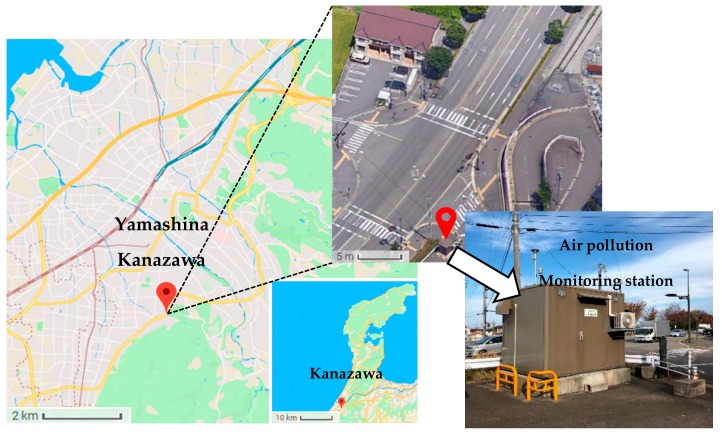
Sampling site at a roadside air pollution monitoring station in Kanazawa.

PM_2.5_ sampling was conducted at an air pollution monitoring station (36°31′43.3″N 136°39′06.6″E) in Kanazawa, the capital city of Ishikawa Prefecture with a population of 464,483 until 2018. The station is located on the roadside in Yamashina-machi, as shown in Figure 1. There were no obvious sources of PM_2.5_ near the station except for the traffic emission. The PM_2.5_ samples were collected for one week each in spring (April 24 to 30, 2017), summer (August 21 to 27, 2017), autumn (November 6 to 12, 2017) and winter (February 19 to 25, 2018). Twenty-four-hour PM_2.5_ samples were collected on quartz fiber filters (2500QAT-UP, Pallflex Products, Putnam, CT, USA) using a high-volume air sampler (HV-1000F, Sibata Scientific Technology Ltd., Saitama, Japan) at a flow rate of 1000 L/min starting from 9:00 a.m. The quartz fiber filters were preheated at 600 °C for 4h before sampling to lower their PAHs blank values. The mass of PM_2.5_ on each filter was measured through gravimetric analysis using a microbalance with an accuracy of 0.01mg.

### 2.2. Chemicals

The US Environmental Protection Agency 610 PAH mix, including fluoranthene (Flt), pyrene (Pyr), benz(*a*)anthracene (BaA), chrysene (Chr), benzo(*b*)fluoranthene (BbF), benzo(k)fluoranthene (BkF), BaP, benzo(*ghi*)perylene (BgPe) and indeno(1,2,3-*cd*)pyrene (IDP), was purchased from Supelco Park (Bellefonte, PA, USA). For NPAHs, a standard containing 9-nitroanthracene (9-NA), 3-nitrophenanthrene (3-NPhe), 1-NP, 6-nitrochrysene (6-NC), 7-nitrobenz(*a*)anthracene (7-NBaA) and 6-nitrobenz(*a*)pyrene (6-NBaP) was purchased from Chiron AS (Trondheim, Norway).

Two internal standards for PAH analysis, Pyr-*d*_10_ and BaP-*d*_12_, were purchased from Wako Pure Chemicals (Osaka, Japan). An internal standard for NPAH analysis, 2-fluoro-7-nitrofluorene (FNF), was purchased from Chiron AS (Trondheim, Norway). All other chemicals used in this study were of reagent analytical grade.

### 2.3. Pretreatment and analysis of PAHs and NPAHs

Pretreatment was performed as described in a previous study [17]. Briefly, an 1/8 of each filter (approximately 45 cm^2^) was cut into small pieces and placed in a flask. Pyr-d_10_, BaP-d_12_ and FNF were added to the flask. Then, PAHs and NPAHs were extracted twice through ultrasonication with ethanol/benzene (1:3, v/v). The solution was then washed, once with solutions of sodium hydroxide and sulfuric acid and twice with distilled water. After adding 100 μL dimethyl sulfoxide, the solution was concentrated on a rotary evaporator, and the residue was dissolved in ethanol. Finally, the solution was filtered through a 0.45 μm membrane filter (HLC-DISK13, Kanto Chemical Co., Inc., Tokyo, Japan) and injected into a vial. After pretreatment, the solution was prepared for the analysis.

In this study, for PAHs, Flt, Pyr, BaA, Chr, BbF, BkF, BaP, BgPe and IDP were determined by high-performance liquid chromatography (HPLC) with fluorescence detection, consistent with our previous study [16]. For NPAHs, 9-NA, 3-NPhe, 1-NP, 7-NBaA and 6-NBaP were determined by HPLC with chemiluminescence detection, the details of which are consistent with our previous study [17,23].

### 2.4. Quality Control and Quality Assurance

All filters were measured before and after sampling at a constant temperature (20–25 °C) and humidity (30–45%). Blank filters were analyzed to check for background contamination during transport. There were no target PAHs on the blank filters, indicating that there was no contamination from the transport of blank samples. The recovery and quantification of PAHs and NPAHs were determined by using the internal standards mentioned above. The recovery of PAHs ranged from 70% to 105%, and the recovery of NPAHs ranged from 62% to 101%.

### 2.5. Health Risk Assessment

To assess the health risk posed by PM_2.5_-bound PAHs and NPAHs in the ambient air, the concentrations of 9 PAHs and 1-NP in our study were expressed by using the equivalent concentration of BaP (BaP_eq_). BaP_eq_ was calculated by the following formula:

(1) $$BaP_{eq}=\sum_{i=1}^{n}(c_{i}\times TEF_{i})$$

where C_i_ is the concentration of the ith individual PAH and NPAH and TEF_i_ is the toxic equivalency factor (TEF) of the ith individual PAH and NPAH. The LCR of PAHs and NPAHs via inhalation exposure was calculated as follows:

(2) $$LCR=UR_{BaP}\times BaP_{eq}$$

where UR_BaP_ is the Unit risk of BaP and equals to 1.1 × 10^−6^ (ng/m^3^)^-1^ [24]. The TEF for the PAHs and NPAHs are shown in Table S1 [25,26].

### 2.6. Meteorological Data

The meteorological data for the sampling periods are shown in Table S2. The data, including temperature (°C), precipitation (mm), relative humidity (%) and wind speed (m/s), were obtained from the Japan Meteorological Agency (http://www.jma.go.jp/jma/index.html).

### 2.7. Statistical Analysis

Statistics were performed using SPSS version 25.0 (IBM Co., Armonk, NY, US). The correlations among PAHs, NPAHs and meteorological data were assessed by Spearman analysis. A *p*-value less than 0.05 indicated that the results were statistically significant. In addition, the normality analysis of our PAHs and NPAHs data was assessed by Q-Q plots. And the result was shown in Figure S1.

## 3. Results

### 3.1. Concentrations of PM_2.5_, PAHs and NPAHs

The mean concentrations of PM_2.5_ are shown in Figure S2, PM_2.5_ was highest in spring (21.7 ± 12.1 μg/m^3^), followed by winter (14.2 ± 2.62 μg/m^3^), autumn (12.3 ± 5.08 μg/m^3^) and summer (10.6 ± 4.26 μg/m^3^). In spring, high PM_2.5_ concentrations might have been affected by Asian dust [27]. The yearly mean concentration of PM_2.5_ was 16.1 μg/m^3^, slightly above the Japan air quality standard for the year (15 μg/m^3^). However, the mean level of PM_2.5_ in our study was much lower than that in Beijing, China (89.5 ± 36.7 μg/m^3^) [28].

**Figure 2:**
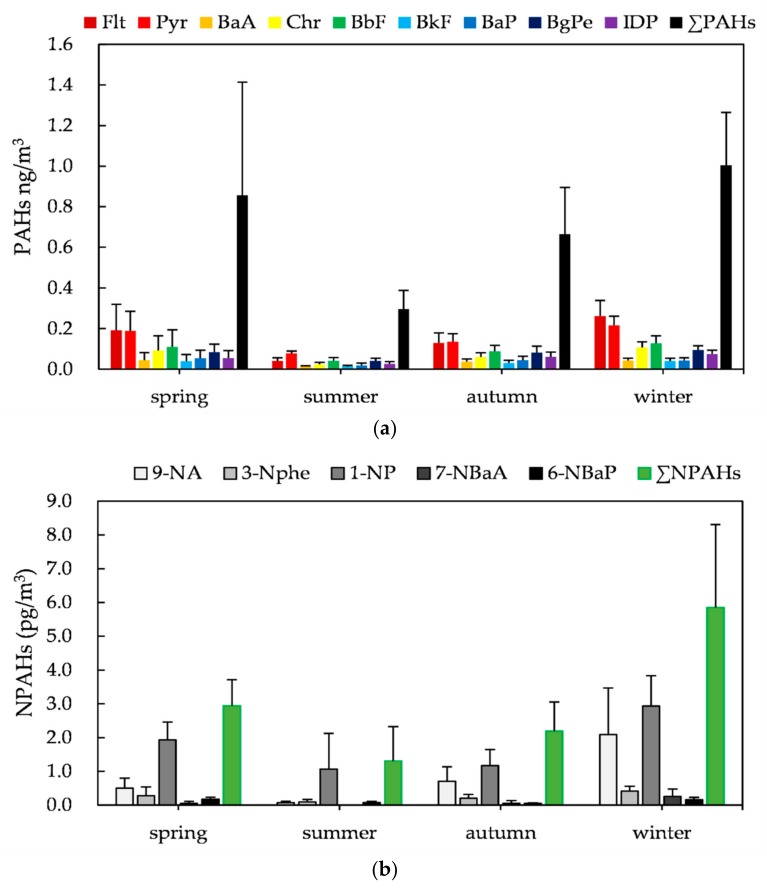
Seasonal variation of atmospheric PAHs and NPAHs: (**a**) PAHs; (**b**) NPAHs.

The atmospheric PAHs and NPAHs in four seasons are shown in Figure 2. The mean concentrations of PAHs were ranked in the following order: winter (1004.6 ± 259.8 pg/m^3^), spring (856.2 ± 557.2 pg/m^3^), autumn (664.7 ± 230.4 pg/m^3^) and summer (296.6 ± 91.1 pg/m^3^). The mean concentrations of NPAHs were ranked in the following order: winter (5.85 ± 2.46 pg/m^3^), spring (2.94 ± 0.77 pg/m^3^), autumn (2.19 ± 0.86 pg/m^3^) and summer (1.30 ± 1.02 pg/m^3^). The mean concentrations of both PAHs and NPAHs were highest in winter and lowest in summer. In this study, the levels of PAHs and NPAHs were much lower than those in our previous study at another monitoring station in Fujie, Kanazawa [19], suggesting that air quality may have improved in recent years.

### 3.2. Composition of PAHs and NPAHs

**Figure 3:**
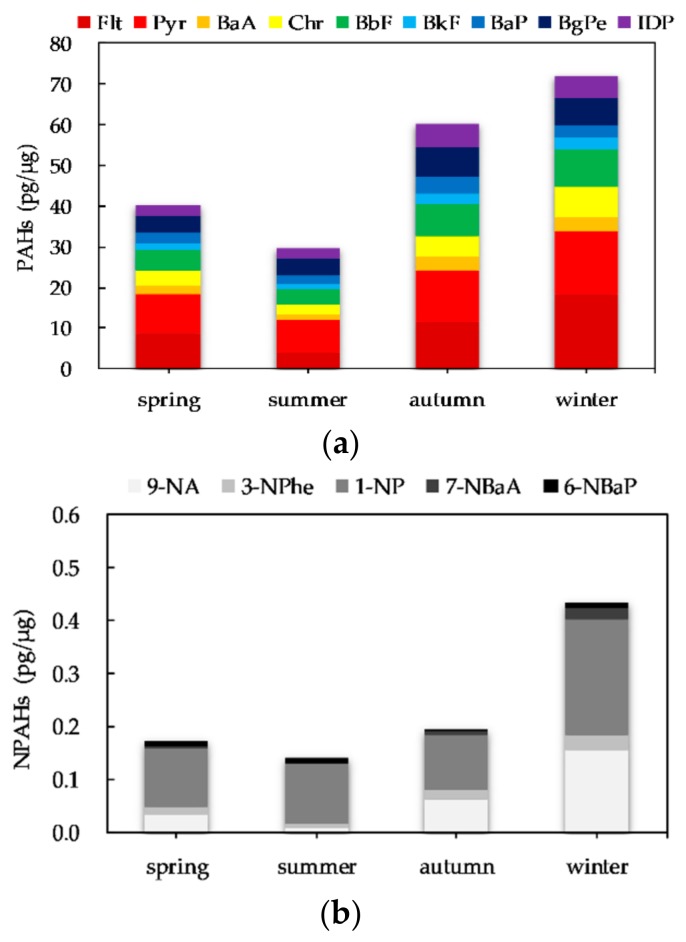
Seasonal variation in composition of PAHs and NPAHs in PM_2.5_: (**a**) PAHs; (**b**)NPAHs.

The seasonal variability in the composition of PAHs and NPAHs in PM_2.5_ is shown in Figure 3. Flt and Pyr were the dominant PAHs in PM_2.5_. In addition, Chr, BbF, BgPe and IDP accounted for a higher proportion in PM_2.5_ than the other PAHs. In PM_2.5_-bound NPAHs, 1-NP was dominant in all four seasons.

**Figure 4:**
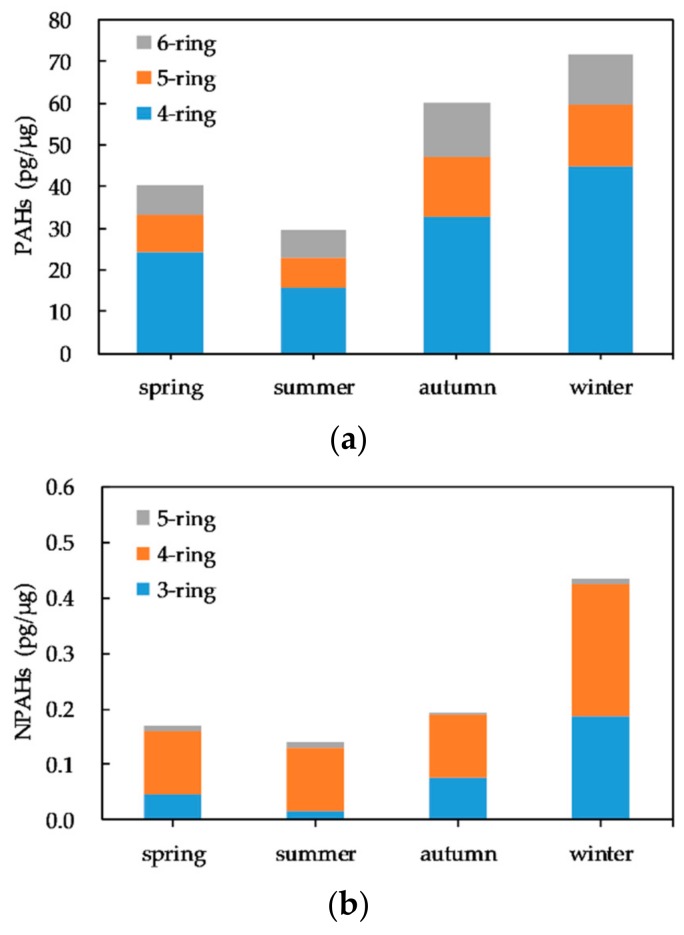
The seasonal variability in the composition of PAHs and NPAHs in PM_2.5_ on the basis of the number of rings: (**a**) PAHs; (**b**)NPAHs. 4-ring PAHs: Flt, Pyr, BaA, Chr; 5-ring PAHs: BbF, BkF, BaP; 6-ring PAHs: BgPe, IDP; 3-ring NPAHs: 9-NA, 3-NPhe; 4-ring NPAHs: 1-NP, 7-NBaA; 5-ring NPAHs: 6-NBaP.

The seasonal variability in the composition of PAHs and NPAHs in PM_2.5_ on the basis of the number of rings is shown in Figure 4. The 4-ring PAHs contributed the most to the total PAHs in the four seasons, accounting for a mean proportion of 57.6%, approximately two- to threefold higher than the proportions of 5-ring (22.8%) and 6-ring (19.6%) PAHs, respectively. The abundance of 4-ring PAHs was highest in winter and lowest in summer possibly because the increased temperature in summer might increase the evaporation of these semi-volatile compounds from the particulate to the gas phase. The composition of 5-ring and 6-ring PAHs exhibited no significant seasonal variation.

The 4-ring NPAHs (1-NP, 7-NBA) were dominant in all seasons. Semi-volatile 3-ring NPAHs (9-NA, 3-NPer) showed similar seasonal variations. The composition and seasonal variations of NPAHs were similar to the observations in our previous study at other monitoring stations in Kanazawa in 2010 [19].

### 3.3. Effect of Meteorological Conditions

Table 1 shows the correlation coefficients (r) for the relationships of PAHs and NPAHs in PM_2.5_ with meteorological data. We did not find any correlations between PAHs, NPAHs with P, RH and WS, which was possibly because our sampling site is very close to the traffic emission sources. However, both PAHs (r = −0.778, *p* < 0.01) and NPAHs (r = −0.626, *p* < 0.01) in PM_2.5_ were significantly and negatively correlated with temperature, which is reflected by a higher concentration in winter than in summer in our study. In winter, all concentrations of PAHs and NPAHs were higher than that in other seasons. Therefore, variations in PAH and NPAH concentrations were not only because the gas-particulate phase partitioning in 4-ring semi-volatile PAHs were caused by variations in temperature [29]; indeed, low-speed driving caused by snowfall in winter might also be a reason for such increased concentrations [21,30].

### 3.4. Concentration ratios of PAHs and NPAHs

The use of DRs is a common conventional method for determining the potential sources of PAHs. According to the different processes producing PAHs, the concentration ratios of PAHs have different characteristics [31]. The advantage of the DRs is that it is less complicated and affords easier results interpretation than other methods [32]. Among the nine target PAHs in our study, the DRs which can be used are (Flt)/((Flt)+(Pyr)) [33], (BaA)/((BaA)+(Chr)) [34], (BaP)/(BgPe) [35], and (IDP)/((IDP)+(BgPe)) [32]. However, DRs were also limited by the uncertainty of the PAHs, which is because 4-ring semi-volatile PAHs perform gas-particulate partitioning due to the variations in temperature [14]. Furthermore, we only did the sampling of PAHs absorbed on the particulate phase in this study. Therefore, we selected (BaP)/(BgPe) and (IDP)/((IDP)+(BgPe)) to verify the accuracy of identifying the traffic emission sources.

Table 2 shows the PAH and NPAH concentration ratios and DRs in our study. A (BaP)/(BgPe) ratio of 0.3–0.4 represents gasoline vehicles, that of 0.46–0.81 represents diesel vehicles and that of 0.3–0.78 represents traffic emissions. The results in our study are consistent with these ranges. Furthermore, most of results were in the range of 0.46–0.81 for diesel vehicles, which indicated that diesel vehicles might be dominant in the mixed traffic source in our sampling site, which is consistent with our previous study [17]. The (BaP)/(BgPe) ratio also seemed to be not only a reliable ratio to identify the traffic source, but also able to distinguish the gasoline and diesel vehicles. However, there was one highest value observed in spring and one lowest value in summer both out of the range of ratio, but the reason for these two special values are unclear and needs further research.

An (IDP)/((IDP)+(BgPe)) ratio of 0.2–0.5 represents gasoline vehicles, and that of 0.38–0.64 represents diesel vehicles, consistent with our results in all four seasons. However, there is a difference with (BaP)/(BgPe), in that the range for diesel vehicles contains that for gasoline vehicles, so (IDP)/((IDP)+(BgPe)) seemed to be unable to distinguish the gasoline and diesel vehicles. However, the ratio (IDP)/((IDP)+(BgPe)) is still effective for identifying mixed traffic emission sources.

The DR of NPAHs used in this study was the ratio (1-NP)/(Pyr), which was selected as an indicator of the contribution of diesel-engine vehicles and coal combustion in our previous studies [16]. The (1-NP)/(Pyr) ratio for diesel-engine vehicles was much higher than that for coal combustion. In this study, (1-NP)/(Pyr) ratios in summer with a mean value of 0.014 and winter (0.014) were far below those in summer (0.04) and winter (0.03) at other monitoring stations in Kanazawa in 2010 [18]. Although the concentration of Pyr has not decreased much, 1-NP has decreased significantly. From 2010 to 2017, the number of vehicles increased, the number of light cars increased by 86% and innovations in engine performance and oil quality caused a decrease in 1-NP. The main reason for these changes was the step-wise restriction of NOx and PM emissions placed on new diesel-engine vehicles by the Japanese government, which is consistent with our previous study [17].

The composition of PAHs and NPAHs in mixed traffic sources is influenced by the driving speed, fuel type and engine conditions [36,37,38]. Therefore, the ratios might vary depending on the traffic characteristics of the sampling site. In our study, the DRs (BaP)/(BgPe) and (IDP)/((IDP)+(BgPe)) were deemed to be reliable ratios to identify the traffic emission sources, and these two ratios might be suitable for Kanazawa or cities with similar emission characteristics as Kanazawa. For example, in Tokyo and Sapporo, the dominant source is traffic emissions, especially diesel vehicles. However, within the method of DRs there still remain some uncertainties. In addition, another study indicated that other specific PAHs (coronene) might be more effective to identify the traffic sources [39]. So, we suggest these DRs should be carefully used to identify the emission sources.

### 3.5. Carcinogenic Risk Assessment

The carcinogenic risk was calculated from the concentrations of nine PAHs and one NPAH (1-NP). The LCR during the sampling period in each season was ranked in the following order: spring (8.98 × 10^−8^), winter (8.29 × 10^−8^), autumn (7.43 × 10^−8^) and summer (3.31 × 10^−8^). Although the concentrations of PAHs and NPAHs were higher in winter than in spring, the LCR in spring was higher than that in winter because the distribution of BaP was higher in spring than in winter. However, the LCR during the sampling period was always significantly below the acceptable limit of cancer risk set by the US EPA (10^−6^). This suggests a low carcinogenic risk posed by PAHs and NPAHs originating from traffic emissions at our sampling site. In addition, the BaPeq (0.062) for PAHs was slightly higher than that in other studies (0.059), which sampled the urban areas of Kanazawa [40] (the calculated BaPeq used the same PAHs and TEF). This suggests a higher carcinogenic risk beside roads than in urban areas. In addition, the LCR of the PAHs in our study was much lower than that in Shenyang [41].

## 4. Conclusions

In this study, PM_2.5_-bound PAHs and NPAHs were sampled at a roadside air pollution monitoring station along the Yamashina 2-chome crossroad of the Yamagawa loop road. We observed seasonal variations in PAHs and NPAHs similar to those in our previous study at a traffic site in Kanazawa. The mean concentrations of PAHs and NPAHs were highest in winter and lowest in summer. In addition, the concentrations of PAHs and NPAHs, especially 1-NP, were lower than those obtained previously at other monitoring stations in Kanazawa, indicating that traffic emission regulations have been enforced effectively. The composition characteristics showed that Flt and Pyr were the dominant PAHs and that 1-NP was the dominant NPAH in the different seasons, all of which were mainly emitted from diesel vehicles. The comparison of PAH concentration ratios in our study with the DRs showed that the ratios (BaP)/(BgPe) and (IDP)/((IDP)+(BgPe)) might be reliable ratios for identifying traffic emission sources. However, the composition characteristics of PAHs and NPAHs from traffic emissions are influenced by the vehicle composition and other related conditions (e.g., driving speed and engine condition), and there are also some uncertainties. Therefore, using DRs for source identification might lead to errors. However, we provide a range of PAH concentration ratios for Kanazawa or cities with similar emission characteristics. The carcinogenic risk assessment showed that PAHs and NPAHs from traffic emissions at our sampling site pose a low cancer risk. However, the carcinogenic properties of some NPAHs are unknown, and the number of detected compounds was limited in our study. Therefore, the health risk to humans needs further research.

## Figures and Tables

**Table 1:** Correlation coefficients for the relationships of PAHs and NPAHs in PM_2.5_ with meteorological data.

Compound	T	P	RH	WS
PAHs	−0.778 **	0.037	0.085	0.059
NPAHs	−0.626 **	0.130	0.325	−0.300

Level of significance: **, *p* < 0.01. T: temperature (°C), P: precipitation (mm), RH: relative humidity (%), WS: wind speed (m/s).

**Table 2:** Concentration ratios of PAHs and NPAHs.

Emission Source	(BaP)/(BgPe)	(IDP)/((IDP)+(BgPe))
Gasoline vehicles	0.3–0.4	0.2–0.5
Diesel vehicles	0.46–0.81	0.35–0.70
Vehicle exhaust	0.3–0.78	
Season		
Spring	0.42–1.02 (0.63)	0.28–0.47 (0.37)
Summer	0.19–0.68 (0.49)	0.34–0.43 (0.39)
Autumn	0.45–0.61 (0.56)	0.39–0.45 (0.43)
Winter	0.34–0.51 (0.48)	0.39–0.49 (0.43)

Data for different seasons are represented as ranges (means).

## Supplementary Materials

The following are available online at https://www.mdpi.com/1660-4601/17/3/805/s1, Figure S1. Q-Q plot diagram of (a) PAHs and (b) NPAHs. Figure S2. Q-Q plot diagram of NPAHs. Table S1: TEF of PAHs and NPAHs. Table S2: Meteorological data during sampling periods.
