# Supplementary material for: Characteristics of PM2.5-Bound Polycyclic Aromatic Hydrocarbons and Nitro-Polycyclic Aromatic Hydrocarbons at A Roadside Air Pollution Monitoring Station in Kanazawa, Japan
Source: Int J Environ Res Public Health. 2020 Jan 28;17(3):805. doi: 10.3390/ijerph17030805 (PMC7037384; doi:10.3390/ijerph17030805)
Supplement: Supplementary file 1 [file ijerph-17-00805-s001.pdf]

Characteristics of $PM_{2.5}$-bound polycyclic aromatic hydrocarbons and nitro-polycyclic aromatic hydrocarbons at a Roadside Air Pollution Monitoring Station, Kanazawa, Japan

Wanli Xing [1], Lulu Zhang [1], Lu Yang [1], Quanyu Zhou [1], Xuan Zhang [1], Akira Toriba [2], Kazuichi Hayakawa [3] and Ning Tang [2,3,*]

[1] Graduate School of Medical Sciences, Kaznazawa University, Kanazawa 920-1192, Japan
[2] Institute of Medical, Pharmaceutical and Health Sciences, Kanazawa University, Kanazawa 920-1192, Japan
[3] Institute of Nature and Environmental Technology, Kanazawa University
* Correspondence: n_tang@staff.kanazawa-u.ac.jp; Tel.: +81-76-234-4455

Figure S1. Q-Q plot diagram of (a) PAHs and (b) NPAHs.

Figure S2. Seasonal variation of $PM_{2.5}$.

Table S1. TEF of PAHs and NPAHs.

Table S2. Meteorological data during sampling periods.

References

24. Nisbet, I.C.; Lagoy, P.K. Toxic equivalency factors (TEFs) for polycyclic aromatic hydrocarbons (PAHs). *Regul. Toxicol. Pharmacol.* **1992**, *16*, 290–300.
25. RIDEM. Rhode Island Air Toxics Guideline. State of Rhode Island Department of Environmental Management. 2008. Available online (accessed on 18 December 2019).

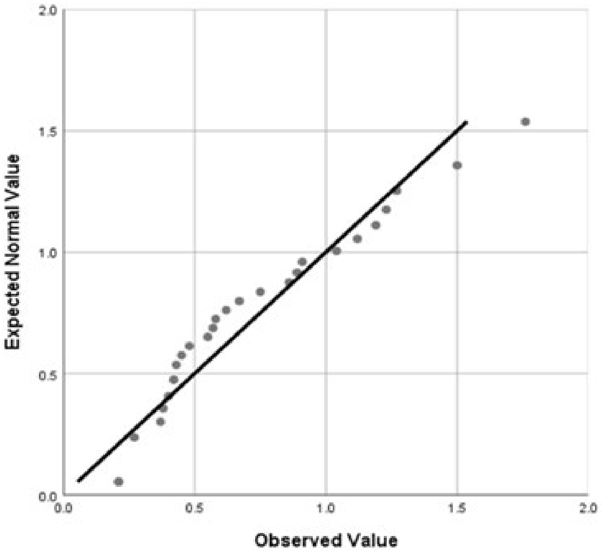

(a)

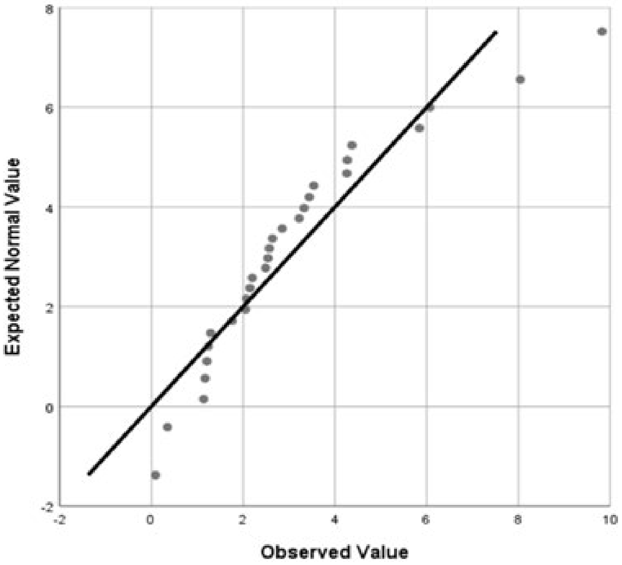

(b)

Figure S1. Q-Q plot diagram of (a) PAHs and (b) NPAHs.

We used the data of atmospheric PAHs and NPAHs for the normality test. As shown in Figure S1, the points of our data are basically coincided with theoretical straight line, which means our data accord with normal distribution. Therefore, we think that our data can represent the actual situation to a certain extent.

Table S1. TEF of PAHs and NPAHs.

Group	Compound	TEF of PAHs [1]	Group	Compound	TEF of NPAHs [2]
PAHs	Flt	0.001	NPAHs	1-NP	0.1
	Pyr	0.001			
	BaA	0.1			
	Chr	0.01			
	BbF	0.1			
	BkF	0.1			
	BaP	1			
	BgPe	0.01			
	IDP	0.1			

[1] [24]; [2] [25].

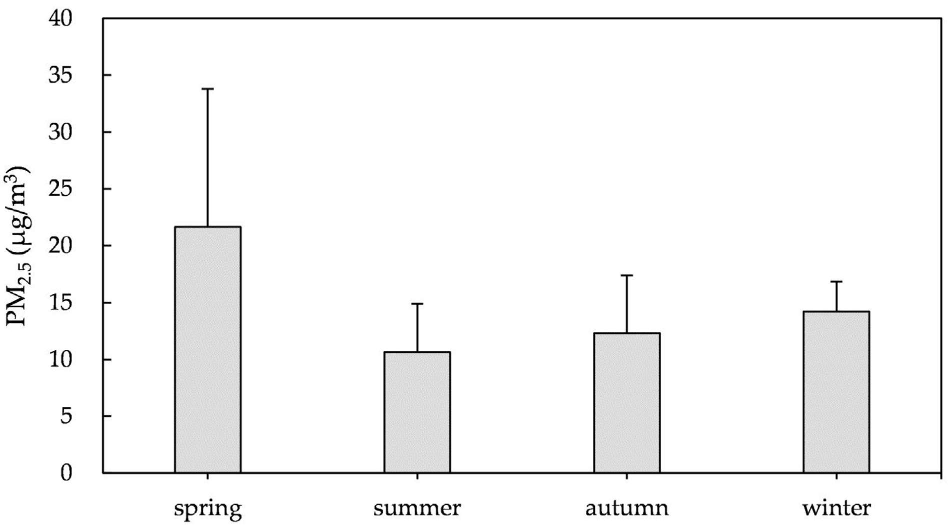

Figure S2. Seasonal variation of $PM_{2.5}$.

Table S2. Meteorological data during sampling periods.

	Date	T (mean, °C)	P (mm)	RH (%)	WS (m/s)
spring	2017.4.24	15.2	--	47.0	2.5
	2017.4.25	18.7	0.0	47.0	3.9
	2017.4.26	14.1	46.5	84.0	3.8
	2017.4.27	12.8	0.5	62.0	3.4
	2017.4.28	15.1	--	45.0	3.7
	2017.4.29	16.2	0.0	52.0	6.1
	2017.4.30	19.7	0.0	38.0	5.9
summer	2017.8.21	28.8	--	69.0	2.4
	2017.8.22	28.9	5.0	66.0	4.0
	2017.8.23	28.4	18.0	77.0	5.8
	2017.8.24	29.9	0.5	67.0	6.7
	2017.8.25	27.9	67.0	76.0	4.2
	2017.8.26	26.6	11.5	62.0	3.1
	2017.8.27	25.7	--	65.0	3.4
autumn	2017.11.6	13.0	--	65.0	2.7
	2017.11.7	14.9	--	63.0	2.4
	2017.11.8	17.9	0.5	66.0	5.3
	2017.11.9	13.1	0.0	60.0	3.9
	2017.11.10	13.3	0.0	53.0	3.1
	2017.11.11	12.7	54.5	63.0	6.8
	2017.11.12	9.7	1.0	65.0	3.3
winter	2018.2.19	2.3	0.0	70.0	1.7
	2018.2.20	4.3	1.5	72.0	3.6
	2018.2.21	2.6	0.0	76.0	2.5
	2018.2.22	2.7	0.5	61.0	3.0
	2018.2.23	4.2	5.0	68.0	4.1
	2018.2.24	4.4	0.5	62.0	4.7
	2018.2.25	3.5	--	63.0	3.1

T: temperature (°C), P: precipitation (mm), RH: relative humidity (%), WS: wind speed (m/s). -- means no available data.
